# Evaluating when (and how) hypertension may be ‘good for your brain’

**DOI:** 10.1093/braincomms/fcad127

**Published:** 2023-04-18

**Authors:** Shino D Magaki, Harry V Vinters

**Affiliations:** Division of Neuropathology, Department of Pathology and Laboratory Medicine, Ronald Reagan UCLA Medical Center and David Geffen School of Medicine, Los Angeles, CA 90095, USA; Division of Neuropathology, Department of Pathology and Laboratory Medicine, Ronald Reagan UCLA Medical Center and David Geffen School of Medicine, Los Angeles, CA 90095, USA; Department of Neurology, Ronald Reagan UCLA Medical Center and David Geffen School of Medicine, Los Angeles, CA 90095, USA; Brain Research Institute, Ronald Reagan UCLA Medical Center and David Geffen School of Medicine, Los Angeles, CA 90095, USA

## Abstract

This scientific commentary refers to ‘Elevated late-life blood pressure may maintain brain oxygenation and slow amyloid-β accumulation, at the expense of cerebral vascular damage’, by Tayler *et al.* (https://doi.org/10.1093/braincomms/fcad112).

This scientific commentary refers to ‘Elevated late-life blood pressure may maintain brain oxygenation and slow amyloid-β accumulation, at the expense of cerebral vascular damage’, by Tayler *et al.* (https://doi.org/10.1093/braincomms/fcad112).

An important and informative study from the Bristol group^1^ published recently in *Brain Communications* provides intriguing data on the possible role of hypertension in maintaining brain oxygenation and modulating the accumulation within the central nervous system (CNS) of amyloid-β protein, thought to be a key element in the progression of brain degeneration associated with dementia. It builds upon many important relevant observations pertinent to brain aging, originating from Professor Love’s group, over several decades. The potential role(s) of amyloid-β—since its discovery by Glenner and Wong in 1984^2^—in the pathogenesis of dementia is not without controversy.^3^ Nevertheless, the accumulation and presence of phospho-tau, amyloid-β and neuritic plaques are three reliable histologic markers that support the autopsy diagnosis of Alzheimer’s disease.^4^ However, removing amyloid-β from the brain does not consistently lead to significant improvement of cognitive decline,^5^ the reasons for which may be extremely complex and multifactorial—including the likelihood that neurodegeneration is well under way prior to amyloid-β deposition within the brain. This study examines how blood pressure may mediate some aspects of its deposition.

The investigation, which uses human autopsy specimens as a starting point, provides a powerful reminder that Alzheimer’s disease is a distinctly human disease—from both clinical and neuropathologic perspectives.^6^ Although elements of Alzheimer’s disease neuropathologic change can be produced (in some cases rather dramatically) in animal models, the constellation of neurocognitive impairment and histopathologic abnormalities (which are believed to contribute to said neurologic disability) occur only in the human brain and result in the synaptic loss characteristic of this form of dementia. Studies based upon autopsies are limited by the fact that the brain specimen can be examined at only one point in time—though in rare instances an opportunity has presented itself to look at autopsy brains when a biopsy has previously been done on the deceased subject.^7^ The brain biopsies done on a small number of the subjects in the Di Patre *et al*. paper were studied by electron microscopy, yielding interesting observations on the microvasculature though, by definition, the ‘*n*’ of samples examined was small.^8^ A morphometric analysis of these same brain biopsies showed evidence of compromise and ‘leakiness’ of the blood–brain barrier (BBB),^9^ which this study seems to support. A more recent quantitative immunohistochemical study^10^ has shown BBB abnormalities in Alzheimer’s disease subjects with varying degrees of cerebral amyloid angiopathy (CAA), especially CAA type 1 in which both capillaries and larger vessels are involved.

The present investigation was based upon both immunohistochemical and biochemical (mainly using enzyme-linked immunosorbent assays, ELISAs) examination of brain samples from subjects with different types or ‘categories’ of dementia, based upon both antemortem clinical examination and neuropathologic study of necropsy brain tissue from affected subjects. Indirect measures suggestive of antemortem brain hypoxia and ischaemia and BBB dysfunction were assessed. Details of the race/ethnicity of the patients whose brains were examined are not provided and might have been of interest, given that the UK represents a culturally and ethnically diverse society. Although it is not specifically indicated in the ‘Materials and Methods’, one assumes that a component of Lewy body disease was excluded from all cases by alpha-synuclein immunohistochemistry on selected tissue blocks. As the authors indicate, their smallest study group comprised those with vascular dementia, and relatively little ‘granularity’ is provided on the neuropathologic findings in those subjects; morphologic substrates of vascular dementia are known to be extremely heterogeneous.^11,12^ It is somewhat surprising that no mention is made of the presence or frequency of brain microbleeds—or any evidence of haemorrhage whatsoever—among the specimens examined (brain microbleeds are strongly associated with CAA, thus by definition with Alzheimer’s disease). The ‘infarcts/ischemic lesions’ described (study cohort section under ‘Materials and Methods’) were probably all (or predominantly) lacunar or microinfarcts rather than larger cystic infarcts. Only half of each brain was examined by histopathology, and accepting the fact that ischaemic brain lesions may be asymmetrical between the cerebral hemispheres (especially in those with vascular dementia), this may have impacted on interpretation of the data in this relatively small group within the larger study.

As the authors acknowledge, a weakness of their study is the reliance (for clinicopathologic correlations) on accurate antemortem blood pressure readings. In the time frame during which the subjects were examined clinically, blood pressure assessments probably evolved from manual to more accurate digital measurements. Per Table 1 in their article,^1^ the blood pressure measurements may have been as few as four over several years, at least in the Alzheimer’s disease and vascular dementia patient groups. Because the study specimens were obtained from a ‘brain bank’ resource, there is no indication that complete autopsies were frequently, or ever, performed on the subjects, so data on generally accepted classic autopsy evidence of hypertension (nephrosclerosis, cardiac/left ventricular hypertrophy) is not available to provide supporting evidence of longstanding high blood pressure. Seminal studies by Lammie *et al*.^13,14^ indeed suggest that ‘hypertensive microvascular small vessel disease’ may, in many cases, be associated with factors other than high blood pressure in ∼30% of patients.

This detailed clinicopathologic investigation represents an excellent step in trying to understand the complex interaction between blood pressure regulation and both normal and pathologic brain aging.

## References

[1] Tayler HM , MacLachlan R, Güzel Ö, Miners JS, Love S. Elevated late-life blood pressure may maintain brain oxygenation and slow amyloid-β accumulation, at the expense of cerebral vascular damage. Brain Commun. 2023;5:fcad112. 10.1093/braincomms/fcad112 PMC10128877 37113314

[2] Glenner GG , Wong CW. Alzheimer's disease: Initial report of the purification and characterization of a novel cerebrovascular amyloid protein. 1984. Biochem Biophys Res Commun. 2012;425:534–539. 22925670 10.1016/j.bbrc.2012.08.020

[3] Serrano-Pozo A , Qian J, Muzikansky A, et al Thal amyloid stages do not significantly impact the correlation between neuropathological change and cognition in the Alzheimer disease continuum. J Neuropathol Exp Neurol. 2016;75:516–526. 27105663 10.1093/jnen/nlw026 PMC6250207

[4] Montine TJ , Phelps CH, Beach TG, et al National Institute on Aging-Alzheimer's Association guidelines for the neuropathologic assessment of Alzheimer's disease: A practical approach. Acta Neuropathol. 2012;123:1–11. 22101365 10.1007/s00401-011-0910-3 PMC3268003

[5] Karran E , De Strooper B. The amyloid hypothesis in Alzheimer disease: New insights from new therapeutics. Nat Rev Drug Discov. 2022;21:306–318. 35177833 10.1038/s41573-022-00391-w

[6] Vinters HV . Emerging concepts in Alzheimer's disease. Annu Rev Pathol. 2015;10:291–319. 25387055 10.1146/annurev-pathol-020712-163927

[7] Di Patre PL , Read SL, Cummings JL, et al Progression of clinical deterioration and pathological changes in patients with Alzheimer disease evaluated at biopsy and autopsy. Arch Neurol. 1999;56:1254–1261. 10520942 10.1001/archneur.56.10.1254

[8] Vinters HV , Secor DL, Read SL, et al Microvasculature in brain biopsy specimens from patients with Alzheimer's disease: An immunohistochemical and ultrastructural study. Ultrastruct Pathol. 1994;18:333–348. 8066824 10.3109/01913129409023202

[9] Stewart PA , Hayakawa K, Akers MA, Vinters HV. A morphometric study of the blood–brain barrier in Alzheimer's disease. Lab Invest. 1992;67:734–742. 1460864

[10] Magaki S , Tang Z, Tung S, et al The effects of cerebral amyloid angiopathy on integrity of the blood–brain barrier. Neurobiol Aging. 2018;70:70–77. 30007166 10.1016/j.neurobiolaging.2018.06.004 PMC6146962

[11] Vinters HV , Zarow C, Borys E, et al Review: Vascular dementia: Clinicopathologic and genetic considerations. Neuropathol Appl Neurobiol. 2018;44:247–266. 29380913 10.1111/nan.12472

[12] Fang C , Magaki SD, Kim RC, Kalaria RN, Vinters HV, Fisher M. Arteriolar neuropathology in cerebral microvascular disease. Neuropathol Appl Neurobiol. 2023;49:e12875. 10.1111/nan.12875 PMC10350910 36564356

[13] Lammie GA , Brannan F, Slattery J, Warlow C. Nonhypertensive cerebral small-vessel disease. An autopsy study. Stroke. 1997;28:2222–2229. 9368569 10.1161/01.str.28.11.2222

[14] Lammie GA . Hypertensive cerebral small vessel disease and stroke. Brain Pathol. 2002;12:358–370. 12146804 10.1111/j.1750-3639.2002.tb00450.x PMC8096033

